# Tumor-Infiltrating Lymphocytes in Head and Neck Cancer: Ready for Prime Time?

**DOI:** 10.3390/cancers14061558

**Published:** 2022-03-18

**Authors:** Alhadi Almangush, Stijn De Keukeleire, Sylvie Rottey, Liesbeth Ferdinande, Tijl Vermassen, Ilmo Leivo, Antti A. Mäkitie

**Affiliations:** 1 Department of Pathology, University of Helsinki, 00014 Helsinki, Finland; 2 Research Program in Systems Oncology, Faculty of Medicine, University of Helsinki, 00014 Helsinki, Finland; antti.makitie@helsinki.fi; 3 Institute of Biomedicine, Pathology, University of Turku, 20520 Turku, Finland; ilmo.leivo@utu.fi; 4 Faculty of Dentistry, Misurata University, 2478 Misurata, Libya; 5 Department of Medical Oncology, University Hospital Ghent, 9000 Ghent, Belgium; stijnj.dekeukeleire@ugent.be (S.D.K.); sylvie.rottey@ugent.be (S.R.); tijl.vermassen@uzgent.be (T.V.); 6 Department of Pathology, University Hospital Ghent, 9000 Ghent, Belgium; liesbeth.ferdinande@ugent.be; 7 Department of Pathology, Turku University Central Hospital, 20521 Turku, Finland; 8 Department of Otorhinolaryngology—Head and Neck Surgery, University of Helsinki and Helsinki University Hospital, HUS, 00029 Helsinki, Finland; 9 Division of Ear, Nose and Throat Diseases, Department of Clinical Sciences, Intervention and Technology, Karolinska Institute and Karolinska University Hospital, 17176 Stockholm, Sweden

**Keywords:** head and neck squamous cell cancer (HNSCC), survival, tumor-infiltrating lymphocytes (TILs)

## Abstract

**Simple Summary:**

The immune response has been shown to be a promising indicator to predict the clinical behavior of many cancers, including head and neck cancer. Tumor-infiltrating lymphocytes (TILs) were widely introduced as an important tool to reveal the status of the immune response. This review discusses the significance of TILs in head and neck cancers.

**Abstract:**

The evaluation of tumor-infiltrating lymphocytes (TILs) has received global attention as a promising prognostic cancer biomarker that can aid in clinical decision making. Proof of their significance was first shown in breast cancer, where TILs are now recommended in the classification of breast tumors. Emerging evidence indicates that the significance of TILs extends to other cancer types, including head and neck cancer. In the era of immunotherapy as a treatment choice for head and neck cancer, assessment of TILs and immune checkpoints is of high clinical relevance. The availability of the standardized method from the International Immuno-oncology Biomarker Working Group (IIBWG) is an important cornerstone toward standardized assessment. The aim of the current article is to summarize the accumulated evidence and to establish a clear premise for future research toward the implementation of TILs in the personalized management of head and neck squamous cell carcinoma patients.

## 1. Introduction

Head and neck squamous cell carcinoma (HNSCC) may manifest in all subsites of the upper aerodigestive tract, including the oral cavity, larynx, oropharynx, and hypopharynx. It is one of the most common cancers worldwide, with 878,348 new cases and 444,347 mortalities in 2020 (https://gco.iarc.fr/today (accessed on 21 October 2021). Unfortunately, the incidence of HNSCC is rising [1]. The main risk factors include tobacco and alcohol consumption (for oral and pharyngo-laryngeal cancers), as well as human papillomavirus (HPV) for oropharyngeal cancer. Regarding the management of HNSCC, new therapeutic perspectives have been demonstrated with the introduction of a minimally invasive surgical approach, using robotic surgery and deintensifying the subsequent chemoradiotherapy [2]. HNSCC is characterized by a high rate of metastatic dissemination, which is associated with worse survival compared with HNSCC cases with no metastasis [3]. While treatment planning relies on traditional criteria, including the TNM staging system (eighth edition [4]) and the status of high-risk HPV, ongoing efforts continue to improve risk stratification. Current tools for risk assessment are cancer-related parameters, while factors related to the tumor microenvironment (TME) are not yet employed in daily practice. It is important to state that the prognostic markers that are widely considered in HNSCC treatment decision making (e.g., tumor size, p16, lymphovascular invasion) are mainly based on evidence from retrospective studies [5,6].

Not surprisingly, the immune response is a major determinant influencing the survival of cancer patients. Moreover, immunotherapy is currently implemented in the standard treatment regimens of recurrent and/or metastatic head and neck cancer patients. However, the evaluation of immunological determinants of a cancer patient is not yet considered in the treatment planning of HNSCC, and there is a need to identify reliable and simple immunological biomarkers to further optimize treatment strategies [7]. Recent research in immuno-oncology and cancer biomarkers has underlined the significance of tumor-infiltrating lymphocytes (TILs) as a promising prognostic indicator in various tumor types, including HNSCC. This paper discusses recent findings regarding TILs in HNSCC and how they can be used to improve individualized treatment.

## 2. Accumulated Evidence on TILs as a Prognosticator: Where Are We Currently?

**Figure 1:**
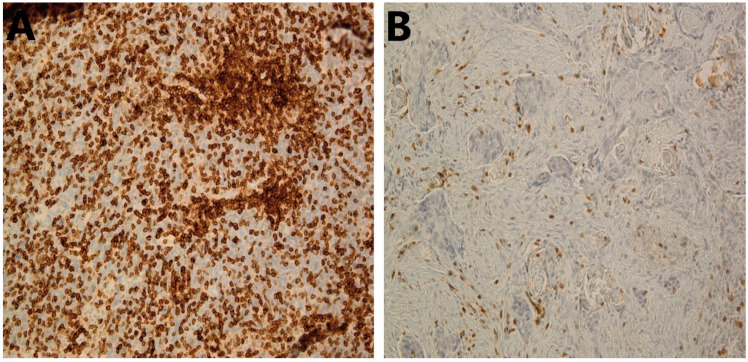
Immunohistochemical staining of CD3^+^ TILs in HNSCC. (**A**) High stromal CD3^+^ TILs in HNSCC tumor; 20×. (**B**) Low stromal CD3^+^ TILs in HNSCC tumor; 20×. HNSCC, head and neck squamous cell carcinoma; TILs, tumor-infiltrating lymphocytes.

**Figure 2:**
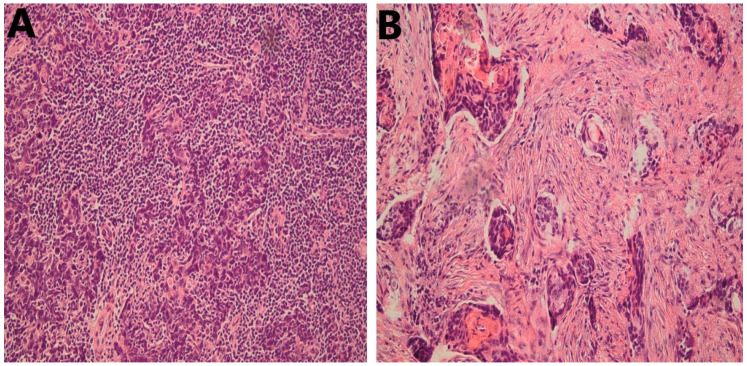
Representative cases of HNSCC stained with HE; 20×. (**A**) High infiltration of stromal TILs; (**B**) Low stromal TILs; 20×. HNSCC, head and neck squamous cell carcinoma; HE, hematoxylin and eosin; TILs, tumor-infiltrating lymphocytes.

Evidence on the prognostic significance of TILs in patients with HNSCC has been rapidly accumulating in recent years. The selection of the molecule, e.g., CD3 (Figure 1), to be analyzed or relying on an overall assessment of TILs (Figure 2) without specific immunostaining is still a point of discussion, and there is no definitive conclusion. On the other hand, the recently published guidelines of the International Immuno-oncology Biomarker Working Group (IIBWG) [8,9] have been utilized in several studies, forming an essential step towards a standardized assessment method and implementation of TILs in routine pathology reporting. Of note, the introduced guidelines have been successfully used in head and neck cancer studies that assessed TILs in hematoxylin and eosin (HE)-stained slides [10,11,12,13].

In addition to many studies that have reported the significance of the overall score of TILs using HE-stained slides in head and neck cancers [10,11,12,13,14,15,16,17,18], the prognostic significance of cell-specific immune markers such as CD3 [19], CD4 [19,20], CD8 [20,21,22,23,24], CD56 [22], CD57 [25], CD163 [26], and FoxP3 [20] has been reported in these cancers. Of note, intensive research on the significance of TILs in HNSCC has been performed, which has led to the accumulation of evidence in systematic reviews and meta-analyses (Table 1). As an example, de Ruiter et al. [27], in their recent meta-analysis (2017), reported a favorable prognostic value of CD3, CD8, and FoxP3 infiltration in HNSCC based on a pooled analyses of good-quality studies with the majority of them having a low risk of bias. Furthermore, in a meta-analysis published in 2020 [28], Bisheshar et al. found a favorable prognostic value for CD56 and CD57. In another recent meta-analysis (2021) on another two TIL subsets, Borsetto et al. [29] found that high CD4 and high CD8 were associated with a reduced risk of death of HNSCC. Of note, when Borsetto et al. [29] conducted meta-analyses for subsites of HNSCC, they found that CD8 was associated with survival of oropharyngeal and hypopharyngeal cancers, but no significant association was found with oral or laryngeal SCC. Focusing on PD-L1, however, Yang et al. (2018) found no significant value for its expression in HNSCC [30]. It is important to explain that the quality of the included studies in these meta-analyses was assessed using the Quality in Prognosis Studies (QUIPS) tool in two of these meta-analyses [28,30] or by the Newcastle–Ottawa Scale in another meta-analysis [29]. Another note is that due to the overlapping time periods in the database search, some studies have been included in each of these meta-analyses [27,29].

Further, meta-analyses for specific subsites of HNSCC have also been conducted in other articles. In laryngeal SCC, for example, a recent meta-analysis (2021) found that TILs in the TME are a reliable prognostic marker [31]. This meta-analysis included studies considering the overall assessment of TILs in HE-stained sections and using subsets of TILs, including CD8 and/or CD3/CD4 [31]. However, the small number of studies (<10 studies) that were included in each meta-analysis, and the small number of cases (<100 patients) in many of the included studies, have been highlighted as a shortcoming [31].

## 3. Clinical Scenarios of TILs in HNSCC

The immune infiltrate, due to its clinical significance, can form a useful additional prognostic parameter. In HNSCC, the assessment of TILs can aid in guiding the patient’s management in two potential clinical scenarios: the first is to contribute to an improved classification of HNSCC based on the TNM–Immune staging system [32]. The currently used tumor-node-metastasis (TNM) classification has been criticized, as many cases will show variable clinical outcomes within the same stage. The addition of the p16-status, a surrogate marker for HPV-induced oropharyngeal cancer, improved risk stratification in HNSCC, but the TNM system requires further refinement. The incorporation of TILs, as an immune parameter in the TNM–Immune system could augment the prognostic performance of the classification and aid in decision making and treatment planning [33].

The second clinical scenario is to serve as an immune classifier to assess the potential need (and subsequently to predict the response) to an immunotherapy regimen [34]. This has already been introduced via the assessment of PD-L1-expressing tumor cells and immune cells in patients with recurrent/metastatic HNSCC [35]. To this extent, several clinical trials are investigating the role of immune checkpoint inhibitors (ICI) for curative approaches, such as the clinical use of neoadjuvant preoperative immunotherapy or administering ICI during concomitant radio(chemo)therapy or as a maintenance/adjuvant therapy [36,37]. To date, however, selecting HNSCC patients who might benefit from immunotherapy remains challenging. The currently reported prognostic factors do not evaluate the full immune status of the patients, as they may be biased by pre-analytical (tissue quality) and spatio-temporal heterogeneity (specimen type and sampling time-point). Subsequently, the expected response to immune-based therapies remains unconsidered in pathology reports of HNSCC. The assessment of TILs on HE-stained slides may provide a useful parameter in addition to traditional prognostic parameters that do not assess the immune response. However, the evaluation of TILs on HE slides should be further investigated in a prospective fashion using standardized methodology, as proposed by the IIBWG, to fully comprehend their function in tailoring treatment with ICI. It will be of high clinical significance to consider the assessment of TILs following the IIBWG criteria in HE-stained sections from ongoing clinical trials of HNSCC [38]. Such a dataset is suitable to be used in the validation of TILs as a prognostic marker [39].

### 3.1. TILs as Indicator for Selection of HNSCC Patients for Immunotherapeutic Approaches

Numerous isolated methods for histological quantification of TILs subsets have been described, each having its own unique scoring technique, pharmacodiagnostic monoclonal antibody, and gradation or cutoff. Despite these differences in methodology, the literature concurs that TILs have an important prognostic value. Their predictive role, however, needs to be further elucidated, and few reports have made contributions regarding this topic. Essentially, tumors can be subdivided in an immune-inflamed or non-inflamed phenotype, as described in the hallmarks of cancer [40]. A preserved immunity is characterized by an adequate amount, diversity, and functioning of immune cells recruited from both the innate and adaptive immune systems, which is required to benefit from treatment with immune checkpoint inhibitors. During the process of tumoral progression, a proportion of transformed cells will not survive, allowing antigen-presenting cells to detect and pick up tumor-related antigens from dead neoplastic cells. Through human leukocyte antigen molecules, these antigens are presented to (CD4^+^ or CD8^+^) T-cells and activate the well-known cascade of tumor-cell recognition, activation, and expansion of effector cells that will induce a tumor-specific immune response. Indeed, inflamed tumor phenotypes may benefit from an improved immune-mediated elimination of tumor cells [41,42]. In anti-CTLA-4-treated melanoma patients, TILs density was significantly increased from baseline in therapy-responders, confirming their predictive significance to ICI [43]. When applied to HNSCC, Mandal et al. [44] reported that an increased density of immune-infiltrating cells, specifically CD56^+^ NK cells, was correlated with a better overall response rate (ORR) in patients treated with a variety of ICI. Furthermore, Hanna et al. [45] reported that HNSCC-patients with a high CD8^+^ lymphocyte rate and PD-1 expression were correlated with improved response rates with anti-PD-1/PD-L1 agents. At present, immunohistochemistry (IHC) for PD-L1 expression is the sole predictive biomarker to determine eligibility for treatment with ICI, yet it lacks robustness. Bearing the aforementioned theory in mind, the evaluation of TILs may provide a useful, additional parameter to assess tumor immune response, which requires further examination in prospective studies using standardized methodology.

### 3.2. The Role of TILs in the Elucidation of De-Escalation Therapies in HNSCC

De-escalation strategies are currently being considered in several malignancies. The goals of de-escalation are reducing therapy-related toxicity, increasing or maintaining survival outcomes, and improving patients´quality of life [46]. Indeed, the multimodal therapeutic approaches applied in HNSCC, including surgery, radiotherapy, and/or chemotherapy, are correlated with both acute and long-term toxicity. Selecting patients for de-escalation may depend on several prognostic biomarkers, such as histopathological characteristics including grade, lymphovascular, or perineural invasion; molecular markers (p16/HPV status); or clinical risk stratification (TNM, performance status) [47]. The emergence of HPV as an etiological factor has served as an important prognosticator in oropharyngeal carcinoma for many years. However, applying de-escalation strategies based on this biomarker has not shown expected benefits with regard to survival [48,49]. The question, therefore, arises if morphological characteristics of the tumor microenvironment, i.e., the immune infiltrate, should be applied during risk stratification as an alternative for HPV status. A recent study by the group of Sylvie Rottey [13] applied the IIBWG method and reported that the increased infiltration of mononuclear cells in oropharyngeal squamous cell carcinoma (OPSCC) correlated with superior survival in comparison to OPSCC with a low TIL density. This outcome was independent of p16-status. Moreover, prognostic stage (AJCC) and stromal TILs density were considered as the two major independent prognostic factors for overall survival, indicating that OPSCC might indeed benefit from a TNM–Immune classification. The AJCC TNM, the eighth edition, deserves credit for introducing a separate classification for p16^+^ OPSCC, though it has also been subjected to criticism for failing to incorporate pivotal scientific evidence regarding the tumor’s immune microenvironment. As the era of immunomodulatory agents is rapidly progressing, immune-related features such as TILs should be added to further improve the clinical and pathological staging, thus aiding physicians in clinical decision making [50]. This concept has been proposed in a similar fashion in colorectal cancer via the introduction of the Immunoscore [51]. According to this methodology, immune cell density is assessed per patient using a digital-pathology-based assay based on the quantification of CD3^+^ and CD8^+^ lymphocytes at the invasive border and tumor core. Patients with high Immunoscores in both areas were associated with better outcomes [52,53]. Although several studies have confirmed the prognostic value of this method in HNSCC, only post hoc analyses and subgroup identification will fully identify its clinical significance [12,13].

## 4. Automated Analysis of TILs

Digital pathology has been used in recent studies to assess markers for HNSCC. Among these, TILs identified by immunohistochemical staining of specific molecules (e.g., CD3, CD4, and CD8) were assessed using an automated method in HNSCC [21,54,55,56,57]. Similarly, the automated signature of CD8xPD-L 1 has been reported as a predictive marker in non-small-cell lung cancer patients [58]. Further research is still warranted to reach the proper application and use of digital analysis tools in our daily practices [59]. The concordance between manual and computational scoring of TILs scores was not excellent in a recent report [60]. However, the digital assessment of TILs in HE-stained slides has been successfully reported in HNSCC [61,62] and showed a superior prognostic performance in a recent large study of HNSCC [63]. These findings have been supported by studies on breast cancer [64] and colorectal cancer [65], where automated analysis showed success in the assessment of TILs.

The findings based on digital analysis can be best applied by considering a semiautomated method, where a pathologist first selects the area to be analyzed to ensure the evaluation of the representative field. The computer application/software will then identify and provide counting/estimation of TILs in the selected area, as was demonstrated in a recent study of oral SCC [55]. This will ensure correct, representative, and reproducible assessment of TILs in the stromal area adjacent to the tumor nests and thus exclude areas outside the tumor border as well as those with necrosis. Further, an open-source algorithm for the automated evaluation of TILs has been recently introduced for melanoma [66]. A similar digital evaluation approach should be considered for TILs in HNSCC to allow the comparison of different analyses.

## 5. Morphological Pitfalls in the Assessment of TILs in HNSCC

**Figure 3:**
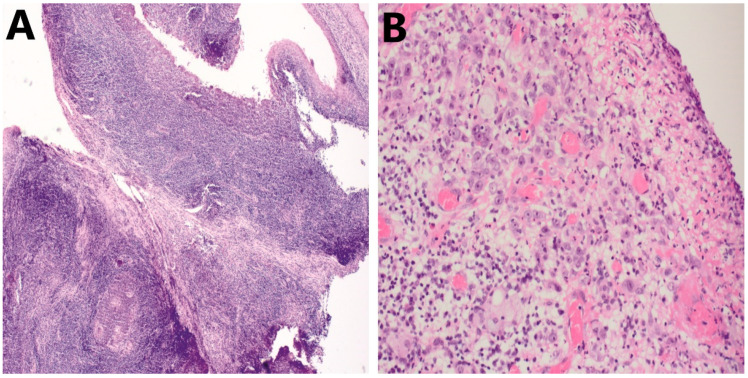
Examples of pitfalls during assessment of TILs in HNSCC on HE. (**A**) Presence of pre-existing lymphoid tissue in HNSCC; 10× (**B**) Presence of ulcerations seeded with polymorphonuclear cells (granulocytes); 20×. HNSCC, head and neck squamous cell carcinoma; TILs, tumor-infiltrating lymphocytes.

Duringthe assessment of TILs, there are two major morphological pitfalls to be distinguished:

(1). Differentiating TILs from a pre-existing immune infiltrate and other immune cells. First, this can be attributed due to the presence of pre-existing lymphoid tissue (Figure 3A), not only in the oropharyngeal/tonsillar region but in all (sub)sites of the head and neck region. Therefore, distinguishing tumor-attracted from pre-existing mononuclear cells may be challenging. Second, regions with ulcerations (Figure 3B) or erosions are seeded with infiltrating immune cells that are predominantly polymorphonuclear cells, which can also hamper the correct assessment of TILs in the TME. Evidently, these should be excluded from evaluation.
(2). Inadequate tumor material for evaluation. In HNSCC, available tissue specimens typically comprise diagnostic biopsies. However, these are often characterized by insufficient amounts of stroma, disabling the correct quantification of stromal lymphocytes in this compartment. Ideally, whole-tumor resected specimens are the ‘golden standard’ to perform quantification of TILs but are mostly unavailable for patients diagnosed with recurrent and unresectable or metastasized HNSCC. In addition, long time intervals may exist between the initial biopsy or resection and the decision to commence palliative treatment. The temporal variance that has occurred in the TME will ultimately have an effect on the use of TILs as a predictive biomarker in this setting.

## 6. Why Is the Assessment of TILs Not Yet Used in the Daily Practice of HNSCC Pathology Reporting?

This question can be properly addressed by considering the limitations and shortcomings of the published studies. First, there are some heterogeneities, such as the variability in the chosen immunohistochemical molecules/markers to assess TILs (e.g., CD3, CD8, FOXP3) in addition to the overall assessment of TILs in HE stained slides. Of note, a recent study in breast cancer demonstrated a better inter-pathologist concordance for the overall assessment of TILs than for the quantification of cell-specific molecules [67]. This issue needs to be studied in HNSCC to find out whether the overall assessment of TILs is more advantageous compared with the identification of the best immune molecule that can be evaluated to aid in clinical decision making as an immune classifier.

Second, there are differences in the cutoff points used to define low, intermediate, and high levels of TILs. The IIBWG recommended scoring the percentage of TILs as a continuous parameter because there is no pre-determined cutoff point for each cancer type [8]. In HNSCC, this issue will need to be studied, ideally for each subsite separately. The histological location of assessment has an obvious impact: stromal TILs have been assessed in most of the published studies and were associated with survival. However, a few studies found intra-tumoral TILs to have significant clinical relevance, yet this quantification method is regarded as challenging [10,22]. The stratification of immune cells in the central area of the tumor and at the invasive margin is therefore advised, as indicated in colon cancer [68]. Scoring TILs at the invasive front has the most relevance with a superior prognostic value compared to TILs scored in the central tumor area of oral cancer [12,22].

Finally, the prognostic significance of TILs in preoperative biopsies of HNSCC has not yet been widely studied. The potential of using TILs in preoperative prognostication to predict response to neoadjuvant therapy can be a valuable step towards personalized treatment. In some cancers, biopsies have been successfully used to assess TILs as a predictor of treatment response [69,70,71] and to predict lymph node metastasis [72]. Similar studies in preoperative HNSCC biopsies are warranted. Of note, a high concordance between biopsies and tumor resection samples with regard to the density of TILs assessed in HE-stained slides has been recently reported in oropharyngeal cancer [57,73]. Unfortunately, the above-mentioned morphological pitfalls that might present during the assessment of TILs (including small biopsy specimens and/or the presence of pre-existing lymphoid tissue) still form a serious obstacle that makes the evaluation of TILs sometimes challenging.

## 7. Future Considerations for the Inclusion of TILs in Daily Clinico-Pathological Practice

In the future, more large-scale prospective studies are needed. Firstly, registry trials using standardized protocols are recommended to optimally describe the TME at the start of systemic therapy, either (neo-)adjuvant or palliative. Based on these registry trials, validation studies in specific settings are important in order to confirm such findings, establish reference values, and ultimately implement TILs in the routine pathology of this cancer type. Subsequently, external quality control of the methodology should be arranged in all participating centers, as performed for several other biomarkers. An inter-laboratory comparison or ‘Ring’ study could therefore be invaluable in this setting: both sensitivity and specificity of the biomarker are validated in different laboratories, mostly coordinated by a main laboratory. Repeatability, inter-laboratory, and inter-observer variability of the technique are tackled in such a trial. Additionally, the dissemination of the quantification technique should be of high priority by teaching the assessors via academic courses and workshops in (inter)national congresses and symposia. Lastly, evaluating TILs as a di- or trichotomized categorical variable via universally set cutoffs would further facilitate the clinical implementation of the quantification, as these will be more easily accepted and interpreted in daily clinical practice compared to the continuous use of TILs. An overview of future steps to be tackled prior to clinical implementation is given in Table 2.

## 8. Conclusions

The accumulated evidence from many studies indicates that TILs are easily estimated in routine HE-stained slides of different subsites of HNSCC and therefore can pave the way towards implementation into daily practice. A recent validation study (2022) has reported that evaluation criteria from IIBWG can be easily used to score TILs in HE sections of oropharyngeal cancer and identify tumors with a high risk of poor survival [74]. Indeed, more validation studies in other subsites of the head and neck need to be considered. Meanwhile, however, the overall assessment of TILs using HE can be already reported in the daily practice of pathologists following the IIBWG method to inform clinicians about the status of the adaptive immune response and to be included in a prognostic algorithm of multiple markers (including TILs) to reach a personalized treatment strategy.

Published findings on specific molecules (e.g., CD3, CD8) are of high clinical relevance and need to be confirmed in large homogenous comparative analyses (i.e., a similar protocol of staining including concentration, cutoff points, risk categorization, etc.). Homogenous cohorts with regard to subsites and stages of HNSCC are also important to compare results. The lack of such homogenous and large cohorts for the validation of these immune molecules makes them emerging biomarkers that are not yet ready for use. Finally, the semiautomated assessment of TILs can be a step toward the precise assessment and reduction in inter-observer variation, if any.

## Figures and Tables

**Table 1:** Summary of meta-analyses on TILs in HNSCC.

Author (Year)	TILs Subset	No. Studies (No. Cases)	Endpoint	SCC Location	Pooled HR (95% CI), *p*
de Ruiter et al. [27] (2017)	CD3+	4 studies (904 cases)	OS	HNSCC	HR 0.64 (0.47–0.85)
		4 studies (735 cases)	DFS	HNSCC	HR 0.63 (0.49–0.82)
	CD8+	9 studies (1697 cases)	OS	HNSCC	HR 0.67 (0.58–0.79)
		7 studies (1053 cases)	DFS	HNSCC	HR 0.50 (0.37–0.68)
		4 studies (596 cases)	LRC	HNSCC	HR 0.82 (0.70–0.96)
	CD4+	4 studies (775 cases)	OS	HNSCC	HR 0.76 (0.64–0.89)
		3 studies (513 cases)	LRC	HNSCC	HR 0.81 (0.68–0.96)
	FoxP3+	6 studies (977 cases)	OS	HNSCC	HR 0.80 (0.70–0.92)
Yang et al. [30] (2018)	PD-L1	21 studies (2477 cases)	OS	HNSCC	HR 0.98 (0.71–1.37), *p* = 0.93
		7 studies (1001 cases)	DFS	HNSCC	HR 1.07 (0.68–1.70), *p* = 0.76
		4 studies (592 cases)	DSS	HNSCC	HR 0.90 (0.63–1.29), *p* = 0.56
		6 studies (585 cases)	PFS	HNSCC	HR 0.71 (0.55–0.93), *p* = 0.01
Bisheshar et al. [28] (2020)	CD56/CD57	4 studies (420 cases)	OS	HNSCC	HR 0.19 (0.11–0.35)
Borsetto et al. [29] (2021)	CD4+	3 studies (548 cases)	OS	HNSSCC	HR 0.77 (0.65–0.93)
	CD8+	6 studies (1220 cases)	OS	HNSSCC	HR 0.64 (0.47–0.88)
	CD4+	3 studies (498 cases)	OS	OPSCC	HR 0.52 (0.31–0.89)
	CD8+	3 studies (326 cases)	OS	OPSCC (HPV-Neg)	HR 0.39 (0.16–0.93)
	CD8+	6 studies (661 cases)	OS	OPSCC (HPV-Pos)	HR 0.40 (0.21–0.76)
	CD8+	3 studies (250 cases)	OS	Hypopharyngeal SCC	HR 0.43 (0.30–0.63)
Rodrigo et al. [31] (2021)	HE staining	4 studies (719 cases)	OS	Laryngeal SCC	HR 0.57 (0.36–0.91), *p* = 0.02
	HE staining	4 studies (659 cases)	DFS	Laryngeal SCC	HR 0.56 (0.34–0.94), *p* = 0.03
	CD8+	5 studies (536 caess)	OS	Laryngeal SCC	HR 0.62 (0.40–0.97), *p* = 0.04
	CD8+	4 studies (574 caess)	DFS	Laryngeal SCC	HR 0.73 (0.60–0.90), *p* = 0.002
	CD3+/CD4+	4 studies (369 caess)	OS	Laryngeal SCC	HR 0.38 (0.16–0.9), *p* = 0.03
	CD3+/CD4+	2 studies (224 caess)	DFS	Laryngeal SCC	HR 0.23 (0.10–0.53), *p* = 0.0005

Abbreviations in Table 1: CI: Confidence interval; DFS: Disease-free survival; DSS: Disease-specific survival; HNSCC: Head and neck squamous cell carcinoma; HR: Hazard ratio; LRC: Locoregional control; OS: Overall survival; PFS: Progression-free survival; TILs: Tumor-infiltrating lymphocytes.

**Table 2:** Summary of future steps needed prior to clinical implementation.

To do	How?	Current Status	Future Steps
Construct standardized methods	International working groups	IIBWG method Immunoscore	Further improvement upon external feedback
Registry trials	Large scale observational trials per subsite	Retrospective trials only	Nation-wide/international analysis using standardized protocols
Prospective validation (predictive/prognostic value)	Clinical trials (introduce TNM–Immune)	Retrospective trials only	Interventional clinical trials(de-escalation strategies)
Determining clinically valuable cutoff	Further dissemination and implementation of TIL quantification	Continuous variable only for more accurate statistical analysis	Introduce di-/trichotomized cutoffs (non vs. moderately vs. highly inflamed tumors)
Education	Symposia Congresses Workshops	Word-of-mouth marketing	Further implementationof the method
Quality control	Ring trials	Non-existing in HNSCC	Further implementationof the method

IIBWG, International Immuno-oncology Biomarker Working Group; HNSCC, head and neck squamous cell carcinoma; TIL, tumor-infiltrating lymphocyte.

## References

[1] Thompson-Harvey A., Yetukuri M., Hansen A.R., Simpson M.C., Adjei Boakye E., Varvares M.A., Osazuwa-Peters N. (2020). Rising incidence of late-stage head and neck cancer in the United States. Cancer.

[2] Meccariello G., Maniaci A., Bianchi G., Cammaroto G., Iannella G., Catalano A., Sgarzani R., De Vito A., Capaccio P., Pelucchi S. (2022). Neck dissection and trans oral robotic surgery for oropharyngeal squamous cell carcinoma. Auris Nasus Larynx.

[3] Pisani P., Airoldi M., Allais A., Aluffi Valletti P., Battista M., Benazzo M., Briatore R., Cacciola S., Cocuzza S., Colombo A. (2020). Metastatic disease in head & neck oncology. Acta Otorhinolaryngol Ital..

[4] Amin M.B., Edge S., Greene F., Byrd D.R., Brookland R.K., Washington M.K., Gershenwald J.E., Compton C.C., Hess K.R., Sullivan D.C. (2017). AJCC Cancer Staging Manual.

[5] Lydiatt W.M., Patel S.G., O’Sullivan B., Brandwein M.S., Ridge J.A., Migliacci J.C., Loomis A.M., Shah J.P. (2017). Head and Neck cancers-major changes in the American Joint Committee on cancer eighth edition cancer staging manual. CA Cancer J. Clin..

[6] Johnson D.E., Burtness B., Leemans C.R., Lui V.W.Y., Bauman J.E., Grandis J.R. (2020). Head and neck squamous cell carcinoma. Nat. Rev. Dis. Primers.

[7] Gavrielatou N., Doumas S., Economopoulou P., Foukas P.G., Psyrri A. (2020). Biomarkers for immunotherapy response in head and neck cancer. Cancer Treat. Rev..

[8] Hendry S., Salgado R., Gevaert T., Russell P.A., John T., Thapa B., Christie M., van de Vijver K., Estrada M.V., Gonzalez-Ericsson P.I. (2017). Assessing Tumor-Infiltrating Lymphocytes in Solid Tumors: A Practical Review for Pathologists and Proposal for a Standardized Method from the International Immuno-Oncology Biomarkers Working Group: Part 2: TILs in Melanoma, Gastrointestinal Tract Carcinomas, Non-Small Cell Lung Carcinoma and Mesothelioma, Endometrial and Ovarian Carcinomas, Squamous Cell Carcinoma of the Head and Neck, Genitourinary Carcinomas, and Primary Brain Tumors. Adv. Anat. Pathol..

[9] Salgado R., Denkert C., Demaria S., Sirtaine N., Klauschen F., Pruneri G., Wienert S., Van den Eynden G., Baehner F.L., Penault-Llorca F. (2015). The evaluation of tumor-infiltrating lymphocytes (TILs) in breast cancer: Recommendations by an International TILs Working Group 2014. Ann. Oncol. Off. J. Eur. Soc. Med. Oncol..

[10] Almangush A., Ruuskanen M., Hagstrom J., Hirvikoski P., Tommola S., Kosma V.M., Nieminen P., Makitie A., Leivo I. (2018). Tumor-infiltrating lymphocytes associate with outcome in nonendemic nasopharyngeal carcinoma: A multicenter study. Hum. Pathol..

[11] Wang Y.Q., Chen Y.P., Zhang Y., Jiang W., Liu N., Yun J.P., Sun Y., He Q.M., Tang X.R., Wen X. (2018). Prognostic significance of tumor-infiltrating lymphocytes in non-disseminated nasopharyngeal carcinoma: A large-scale cohort study. Int. J. Cancer.

[12] Heikkinen I., Bello I.O., Wahab A., Hagstrom J., Haglund C., Coletta R.D., Nieminen P., Makitie A.A., Salo T., Leivo I. (2019). Assessment of Tumor-infiltrating Lymphocytes Predicts the Behavior of Early-stage Oral Tongue Cancer. Am. J. Surg. Pathol..

[13] De Keukeleire S.J., Vermassen T., De Meulenaere A., Deron P., Huvenne W., Duprez F., Creytens D., Van Dorpe J., Rottey S., Ferdinande L. (2021). Tumour infiltrating lymphocytes in oropharyngeal carcinoma: Prognostic value and evaluation of a standardised method. Pathology.

[14] Ward M.J., Thirdborough S.M., Mellows T., Riley C., Harris S., Suchak K., Webb A., Hampton C., Patel N.N., Randall C.J. (2014). Tumour-infiltrating lymphocytes predict for outcome in HPV-positive oropharyngeal cancer. Br. J. Cancer.

[15] Ruangritchankul K., Sandison A., Warburton F., Guerrero-Urbano T., Reis Ferreira M., Lei M., Thavaraj S. (2019). Clinical evaluation of tumour-infiltrating lymphocytes as a prognostic factor in patients with human papillomavirus-associated oropharyngeal squamous cell carcinoma. Histopathology.

[16] Faraji F., Fung N., Zaidi M., Gourin C.C., Eisele D.W., Rooper L.M., Fakhry C. (2019). Tumor-infiltrating lymphocyte quantification stratifies early-stage human papillomavirus oropharynx cancer prognosis. Laryngoscope.

[17] Xu Q., Wang C., Yuan X., Feng Z., Han Z. (2017). Prognostic Value of Tumor-Infiltrating Lymphocytes for Patients With Head and Neck Squamous Cell Carcinoma. Transl. Oncol..

[18] Wang J., Wang S., Song X., Zeng W., Wang S., Chen F., Ding H. (2016). The prognostic value of systemic and local inflammation in patients with laryngeal squamous cell carcinoma. Onco Targets Ther..

[19] Zhang D., Tang D., Heng Y., Zhu X.K., Zhou L., Tao L., Lu L.M. (2021). Prognostic Impact of Tumor-Infiltrating Lymphocytes in Laryngeal Squamous Cell Carcinoma Patients. Laryngoscope.

[20] Wang J., Tian S., Sun J., Zhang J., Lin L., Hu C. (2020). The presence of tumour-infiltrating lymphocytes (TILs) and the ratios between different subsets serve as prognostic factors in advanced hypopharyngeal squamous cell carcinoma. BMC Cancer.

[21] Huang Y., Lin C., Kao H.K., Hung S.Y., Ko H.J., Huang Y.C., Chang Y.L., Chang K.P. (2020). Digital Image Analysis of CD8+ and CD3+ Tumor-Infiltrating Lymphocytes in Tongue Squamous Cell Carcinoma. Cancer Manag. Res..

[22] Caruntu A., Moraru L., Lupu M., Vasilescu F., Dumitrescu M., Cioplea M., Popp C., Dragusin A., Caruntu C., Zurac S. (2021). Prognostic Potential of Tumor-Infiltrating Immune Cells in Resectable Oral Squamous Cell Carcinoma. Cancers.

[23] De Meulenaere A., Vermassen T., Aspeslagh S., Deron P., Duprez F., Laukens D., Van Dorpe J., Ferdinande L., Rottey S. (2017). Tumor PD-L1 status and CD8(+) tumor-infiltrating T cells: Markers of improved prognosis in oropharyngeal cancer. Oncotarget.

[24] De Meulenaere A., Vermassen T., Aspeslagh S., Zwaenepoel K., Deron P., Duprez F., Rottey S., Ferdinande L. (2017). Prognostic markers in oropharyngeal squamous cell carcinoma: Focus on CD70 and tumour infiltrating lymphocytes. Pathology.

[25] Taghavi N., Bagheri S., Akbarzadeh A. (2016). Prognostic implication of CD57, CD16, and TGF-beta expression in oral squamous cell carcinoma. J. Oral Pathol. Med. Off. Publ. Int. Assoc. Oral Pathol. Am. Acad. Oral Pathol..

[26] Hori Y., Kubota A., Yokose T., Furukawa M., Matsushita T., Katsumata N., Oridate N. (2021). Prognostic Role of Tumor-Infiltrating Lymphocytes and Tumor Budding in Early Oral Tongue Carcinoma. Laryngoscope.

[27] de Ruiter E.J., Ooft M.L., Devriese L.A., Willems S.M. (2017). The prognostic role of tumor infiltrating T-lymphocytes in squamous cell carcinoma of the head and neck: A systematic review and meta-analysis. Oncoimmunology.

[28] Bisheshar S.K., De Ruiter E.J., Devriese L.A., Willems S.M. (2020). The prognostic role of NK cells and their ligands in squamous cell carcinoma of the head and neck: A systematic review and meta-analysis. Oncoimmunology.

[29] Borsetto D., Tomasoni M., Payne K., Polesel J., Deganello A., Bossi P., Tysome J.R., Masterson L., Tirelli G., Tofanelli M. (2021). Prognostic Significance of CD4+ and CD8+ Tumor-Infiltrating Lymphocytes in Head and Neck Squamous Cell Carcinoma: A Meta-Analysis. Cancers.

[30] Yang W.F., Wong M.C.M., Thomson P.J., Li K.Y., Su Y.X. (2018). The prognostic role of PD-L1 expression for survival in head and neck squamous cell carcinoma: A systematic review and meta-analysis. Oral Oncol.

[31] Rodrigo J.P., Sanchez-Canteli M., Lopez F., Wolf G.T., Hernandez-Prera J.C., Williams M.D., Willems S.M., Franchi A., Coca-Pelaz A., Ferlito A. (2021). Tumor-Infiltrating Lymphocytes in the Tumor Microenvironment of Laryngeal Squamous Cell Carcinoma: Systematic Review and Meta-Analysis. Biomedicines.

[32] Taube J.M. (2014). Emerging immunologic biomarkers: Setting the (TNM-immune) stage. Clin. Cancer Res..

[33] Almangush A., Bello I.O., Heikkinen I., Hagstrom J., Haglund C., Kowalski L.P., Coletta R.D., Makitie A.A., Salo T., Leivo I. (2021). Improving Risk Stratification of Early Oral Tongue Cancer with TNM-Immune (TNM-I) Staging System. Cancers.

[34] Zandberg D.P., Algazi A.P., Jimeno A., Good J.S., Fayette J., Bouganim N., Ready N.E., Clement P.M., Even C., Jang R.W. (2019). Durvalumab for recurrent or metastatic head and neck squamous cell carcinoma: Results from a single-arm, phase II study in patients with ≥25% tumour cell PD-L1 expression who have progressed on platinum-based chemotherapy. Eur. J. Cancer.

[35] Burtness B., Harrington K.J., Greil R., Soulieres D., Tahara M., de Castro G., Psyrri A., Baste N., Neupane P., Bratland A. (2019). Pembrolizumab alone or with chemotherapy versus cetuximab with chemotherapy for recurrent or metastatic squamous cell carcinoma of the head and neck (KEYNOTE-048): A randomised, open-label, phase 3 study. Lancet.

[36] Masarwy R., Kampel L., Horowitz G., Gutfeld O., Muhanna N. (2021). Neoadjuvant PD-1/PD-L1 Inhibitors for Resectable Head and Neck Cancer: A Systematic Review and Meta-analysis. JAMA Otolaryngol Head Neck Surg..

[37] Machiels J.P., Tao Y., Burtness B., Tahara M., Licitra L., Rischin D., Waldron J., Simon C., Gregoire V., Harrington K. (2020). Pembrolizumab given concomitantly with chemoradiation and as maintenance therapy for locally advanced head and neck squamous cell carcinoma: KEYNOTE-412. Future Oncol..

[38] Lee N.Y., Ferris R.L., Psyrri A., Haddad R.I., Tahara M., Bourhis J., Harrington K., Chang P.M., Lin J.C., Razaq M.A. (2021). Avelumab plus standard-of-care chemoradiotherapy versus chemoradiotherapy alone in patients with locally advanced squamous cell carcinoma of the head and neck: A randomised, double-blind, placebo-controlled, multicentre, phase 3 trial. Lancet Oncol..

[39] Luen S.J., Salgado R., Fox S., Savas P., Eng-Wong J., Clark E., Kiermaier A., Swain S.M., Baselga J., Michiels S. (2017). Tumour-infiltrating lymphocytes in advanced HER2-positive breast cancer treated with pertuzumab or placebo in addition to trastuzumab and docetaxel: A retrospective analysis of the CLEOPATRA study. Lancet Oncol..

[40] Hanahan D., Weinberg R.A. (2011). Hallmarks of cancer: The next generation. Cell.

[41] Bai R., Lv Z., Xu D., Cui J. (2020). Predictive biomarkers for cancer immunotherapy with immune checkpoint inhibitors. Biomark. Res..

[42] Cogdill A.P., Andrews M.C., Wargo J.A. (2017). Hallmarks of response to immune checkpoint blockade. Br. J. Cancer.

[43] Forget M.A., Haymaker C., Hess K.R., Meng Y.J., Creasy C., Karpinets T., Fulbright O.J., Roszik J., Woodman S.E., Kim Y.U. (2018). Prospective Analysis of Adoptive TIL Therapy in Patients with Metastatic Melanoma: Response, Impact of Anti-CTLA4, and Biomarkers to Predict Clinical Outcome. Clin. Cancer Res..

[44] Mandal R., Senbabaoglu Y., Desrichard A., Havel J.J., Dalin M.G., Riaz N., Lee K.W., Ganly I., Hakimi A.A., Chan T.A. (2016). The head and neck cancer immune landscape and its immunotherapeutic implications. JCI Insight.

[45] Hanna G.J., Lizotte P., Cavanaugh M., Kuo F.C., Shivdasani P., Frieden A., Chau N.G., Schoenfeld J.D., Lorch J.H., Uppaluri R. (2018). Frameshift events predict anti-PD-1/L1 response in head and neck cancer. JCI Insight.

[46] Chitsike L., Duerksen-Hughes P.J. (2021). Targeted Therapy as a Potential De-Escalation Strategy in Locally Advanced HPV-Associated Oropharyngeal Cancer: A Literature Review. Front. Oncol..

[47] Denaro N., Russi E.G., Merlano M.C. (2018). Pros and Cons of the New Edition of TNM Classification of Head and Neck Squamous Cell Carcinoma. Oncology.

[48] Rosenberg A.J., Vokes E.E. (2021). Optimizing Treatment De-Escalation in Head and Neck Cancer: Current and Future Perspectives. Oncologist.

[49] Gillison M.L., Trotti A.M., Harris J., Eisbruch A., Harari P.M., Adelstein D.J., Jordan R.C.K., Zhao W., Sturgis E.M., Burtness B. (2019). Radiotherapy plus cetuximab or cisplatin in human papillomavirus-positive oropharyngeal cancer (NRG Oncology RTOG 1016): A randomised, multicentre, non-inferiority trial. Lancet.

[50] Mehanna H., Robinson M., Hartley A., Kong A., Foran B., Fulton-Lieuw T., Dalby M., Mistry P., Sen M., O’Toole L. (2019). Radiotherapy plus cisplatin or cetuximab in low-risk human papillomavirus-positive oropharyngeal cancer (De-ESCALaTE HPV): An open-label randomised controlled phase 3 trial. Lancet.

[51] El Sissy C., Kirilovsky A., Zeitoun G., Marliot F., Haicheur N., Lagorce-Pages C., Galon J., Pages F. (2021). Therapeutic Implications of the Immunoscore in Patients with Colorectal Cancer. Cancers.

[52] Bruni D., Angell H.K., Galon J. (2020). The immune contexture and Immunoscore in cancer prognosis and therapeutic efficacy. Nat. Rev. Cancer.

[53] Galon J., Mlecnik B., Bindea G., Angell H.K., Berger A., Lagorce C., Lugli A., Zlobec I., Hartmann A., Bifulco C. (2014). Towards the introduction of the ‘Immunoscore’ in the classification of malignant tumours. J. Pathol..

[54] De Ruiter E.J., de Roest R.H., Brakenhoff R.H., Leemans C.R., de Bree R., Terhaard C.H.J., Willems S.M. (2020). Digital pathology-aided assessment of tumor-infiltrating T lymphocytes in advanced stage, HPV-negative head and neck tumors. Cancer Immunol. Immunother..

[55] Sung Y.E., Kim M.S., Lee Y.S. (2021). Proposal of a scoring system for predicting pathological risk based on a semiautomated analysis of whole slide images in oral squamous cell carcinoma. Head Neck.

[56] Hartman D.J., Ahmad F., Ferris R.L., Rimm D.L., Pantanowitz L. (2018). Utility of CD8 score by automated quantitative image analysis in head and neck squamous cell carcinoma. Oral Oncol.

[57] De Meulenaere A., Vermassen T., Creytens D., Aspeslagh S., Deron P., Duprez F., Rottey S., Van Dorpe J.A., Ferdinande L. (2018). Importance of choice of materials and methods in PD-L1 and TIL assessment in oropharyngeal squamous cell carcinoma. Histopathology.

[58] Althammer S., Tan T.H., Spitzmuller A., Rognoni L., Wiestler T., Herz T., Widmaier M., Rebelatto M.C., Kaplon H., Damotte D. (2019). Automated image analysis of NSCLC biopsies to predict response to anti-PD-L1 therapy. J. Immunother. Cancer.

[59] Acs B., Salgado R., Hartman J. (2021). What do we still need to learn on digitally assessed biomarkers?. EBioMedicine.

[60] Sun P., He J., Chao X., Chen K., Xu Y., Huang Q., Yun J., Li M., Luo R., Kuang J. (2021). A Computational Tumor-Infiltrating Lymphocyte Assessment Method Comparable with Visual Reporting Guidelines for Triple-Negative Breast Cancer. EBioMedicine.

[61] Shaban M., Khurram S.A., Fraz M.M., Alsubaie N., Masood I., Mushtaq S., Hassan M., Loya A., Rajpoot N.M. (2019). A Novel Digital Score for Abundance of Tumour Infiltrating Lymphocytes Predicts Disease Free Survival in Oral Squamous Cell Carcinoma. Sci. Rep..

[62] Badr M., Johrens K., Allgauer M., Boxberg M., Weichert W., Tinhofer I., Denkert C., Schirmacher P., Stenzinger A., Budczies J. (2019). Morphomolecular analysis of the immune tumor microenvironment in human head and neck cancer. Cancer Immunol. Immunother..

[63] Shaban M., Ahmed Raza S.E., Hassan M., Jamshed A., Mushtaq S., Loya A., Batis N., Brooks J., Nankivell P., Sharma N. (2021). A digital score of tumour-associated stroma infiltrating lymphocytes predicts survival in head and neck squamous cell carcinoma. J. Pathol..

[64] Abe N., Matsumoto H., Takamatsu R., Tamaki K., Takigami N., Uehara K., Kamada Y., Tamaki N., Motonari T., Unesoko M. (2020). Quantitative digital image analysis of tumor-infiltrating lymphocytes in HER2-positive breast cancer. Virchows Arch. Int. J. Pathol..

[65] Yoo S.Y., Park H.E., Kim J.H., Wen X., Jeong S., Cho N.Y., Gwon H.G., Kim K., Lee H.S., Jeong S.Y. (2020). Whole-Slide Image Analysis Reveals Quantitative Landscape of Tumor-Immune Microenvironment in Colorectal Cancers. Clin. Cancer Res..

[66] Acs B., Ahmed F.S., Gupta S., Wong P.F., Gartrell R.D., Sarin Pradhan J., Rizk E.M., Gould Rothberg B., Saenger Y.M., Rimm D.L. (2019). An open source automated tumor infiltrating lymphocyte algorithm for prognosis in melanoma. Nat. Commun..

[67] Nederlof I., De Bortoli D., Bareche Y., Nguyen B., de Maaker M., Hooijer G.K.J., Buisseret L., Kok M., Smid M., Van den Eynden G. (2019). Comprehensive evaluation of methods to assess overall and cell-specific immune infiltrates in breast cancer. Breast Cancer Res. BCR.

[68] Galon J., Costes A., Sanchez-Cabo F., Kirilovsky A., Mlecnik B., Lagorce-Pages C., Tosolini M., Camus M., Berger A., Wind P. (2006). Type, density, and location of immune cells within human colorectal tumors predict clinical outcome. Science.

[69] Miyakita H., Sadahiro S., Suzuki T., Chan L.F., Ogimi T., Okada K., Yamamoto S., Kajiwara H. (2020). Tumor-Infiltrating Lymphocytes in Biopsy Specimens Obtained 7 Days after Starting Chemoradiotherapy for Rectal Cancer Are Predictors of the Response to Chemoradiotherapy. Oncology.

[70] Miyasaka Y., Yoshimoto Y., Murata K., Noda S.E., Ando K., Ebara T., Okonogi N., Kaminuma T., Yamada S., Ikota H. (2020). Treatment outcomes of patients with adenocarcinoma of the uterine cervix after definitive radiotherapy and the prognostic impact of tumor-infiltrating CD8+ lymphocytes in pre-treatment biopsy specimens: A multi-institutional retrospective study. J. Radiat. Res..

[71] Harada Y., Kazama S., Morikawa T., Sonoda H., Ishi H., Emoto S., Murono K., Kaneko M., Sasaki K., Shuno Y. (2021). Clinical significance of CD8(+) and FoxP3(+) tumor-infiltrating lymphocytes and MFG-E8 expression in lower rectal cancer with preoperative chemoradiotherapy. Mol. Clin. Oncol..

[72] Takada K., Kashiwagi S., Asano Y., Goto W., Kouhashi R., Yabumoto A., Morisaki T., Shibutani M., Takashima T., Fujita H. (2020). Prediction of lymph node metastasis by tumor-infiltrating lymphocytes in T1 breast cancer. BMC Cancer.

[73] Brcic I., Gallob M., Schwantzer G., Zrnc T., Weiland T., Thurnher D., Wolf A., Brcic L. (2020). Concordance of tumor infiltrating lymphocytes, PD-L1 and p16 expression in small biopsies, resection and lymph node metastases of oropharyngeal squamous cell carcinoma. Oral Oncol..

[74] Almangush A., Jouhi L., Atula T., Haglund C., Makitie A.A., Hagstrom J., Leivo I. (2022). Tumour-infiltrating lymphocytes in oropharyngeal cancer: A validation study according to the criteria of the International Immuno-Oncology Biomarker Working Group. Br. J. Cancer.

